# High prevalence of syringe lending among HIV-positive people who inject drugs in Bangkok, Thailand

**DOI:** 10.1186/s12954-015-0050-5

**Published:** 2015-06-02

**Authors:** Pauline Voon, Kanna Hayashi, Lianping Ti, Karyn Kaplan, Paisan Suwannawong, Evan Wood, Thomas Kerr

**Affiliations:** Urban Health Research Initiative, British Columbia Centre for Excellence in HIV/AIDS, St. Paul’s Hospital, 608 - 1081 Burrard Street, Vancouver, BC V6Z 1Y6 Canada; School of Population and Public Health, University of British Columbia, Vancouver, BC Canada; Thai AIDS Treatment Action Group, Bangkok, Thailand; Department of Medicine, University of British Columbia, St. Paul’s Hospital, Vancouver, BC Canada

**Keywords:** HIV, Injection drug use, People who inject drugs, Syringe lending, Syringe sharing, Bangkok, Thailand, Polysubstance use, Harm reduction, Antiretroviral

## Abstract

**Background:**

Syringe sharing continues to be a major driver of the HIV pandemic. In light of efforts to enhance access to sterile syringes and promote secondary prevention among HIV-positive individuals, we sought to identify the prevalence and correlates of used syringe lending among self-reported HIV-positive people who inject drugs (PWID) in Bangkok, Thailand.

**Findings:**

We used bivariable statistics to examine factors associated with self-reported syringe lending among self-reported HIV-positive PWID participating in the Mitsampan Community Research Project, a serial cross-sectional study of PWID in Bangkok, between June 2009 and October 2011. In total, 127 individuals were eligible for this analysis, including 25 (19.7 %) women. Twenty-one (16.5 %) participants reported syringe lending in the prior 6 months. Factors significantly associated with syringe lending included daily methamphetamine injection (odds ratio (OR) = 10.2, 95 % CI, 2.1–53.6), daily midazolam injection (OR = 3.1, 95 % CI, 1.1–8.7), use of drugs in combination (OR = 4.5, 95 % CI, 1.0–41.6), injecting with others on a frequent basis (OR = 4.25, 95 % CI, 1.3–18.3), and not receiving antiretroviral therapy (OR = 2.9, 95 % CI, 1.1–7.9).

**Conclusions:**

A high prevalence of syringe lending was observed among self-reported HIV-positive PWID in Bangkok, which was associated with high intensity drug use, polysubstance use, and frequently injecting with others. It is particularly concerning that individuals who lent syringes were more likely to be untreated for HIV disease given the known benefits of antiretroviral provision on the prevention of HIV transmission. These findings underscore the need to expand access to sterile syringes and HIV treatment among HIV-positive PWID in Thailand.

## Introduction

Syringe sharing among people who inject drugs (PWID) continues to be a major driver of the global HIV/AIDS pandemic. Globally, an estimated 1 in 10 new HIV infections is caused by injection drug use [1]. The World Health Organization and other United Nations agencies consider needle and syringe programs (NSPs) to be a crucial component of HIV prevention efforts among PWID [2]. However, in Thailand, despite endorsement of NSPs by public health authorities, such programs continue to be regarded as illegal [3], resulting in NSP coverage as low as <1 % among PWID in Thailand [4].

Despite the legal controversy regarding NSPs, efforts are currently underway to enhance access to sterile syringes and promote secondary prevention among HIV-positive PWID in Thailand, including the provision of sterile syringes within drop-in centers for PWID that are operated by civil society and international organizations [5]. In light of these efforts, we investigated the prevalence and correlates of syringe lending among a community-recruited sample of HIV-positive PWID in Bangkok, Thailand.

## Methods

The Mitsampan Community Research Project is a collaborative research project involving the Mitsampan Harm Reduction Center (Bangkok, Thailand), the Thai AIDS Treatment Action Group (Bangkok, Thailand), Chulalongkorn University (Bangkok, Thailand), and the British Columbia Centre for Excellence in HIV/AIDS/University of British Columbia (Vancouver, Canada). Between June 2009 and October 2011, the research partners undertook a serial cross-sectional study involving 650 unique community-recruited PWID. Potential participants were recruited through peer outreach and word of mouth and were invited to attend the Mitsampan Harm Reduction Center or O-Zone House (local drop-in centers for people who use drugs) to be part of the study. Adults residing in Bangkok or adjacent provinces who had injected drug(s) in the previous 6 months were eligible for participation in the study. All participants provided oral informed consent and completed an interviewer-administered questionnaire eliciting demographic data as well as information about drug use patterns, HIV risk behavior, and experiences with healthcare services. Participants received a stipend of 350 Thai Baht (approximately $11 USD) upon completion of the questionnaire. The study was approved by the research ethics boards at Chulalongkorn University and the University of British Columbia.

The present analysis was restricted to PWID who reported being HIV-positive. The primary outcome of interest was reporting syringe lending in the 6 months prior to the time of interview, as defined by responding “Yes” to the question: “Have you lent your used needles/syringes to someone else in the last six months?” We compared the demographic, behavioral, and social/structural characteristics of PWID who did and did not report syringe lending using Pearson’s *X*^2^ test and Fisher’s exact test (when one or more cells contained values less than or equal to five). Variables considered included the following: median age (<38 years vs. ≥38 years), gender (male vs. female), education level (<secondary education vs. ≥secondary education), incarceration (yes vs. no), frequent heroin injection (≥daily vs. <daily), frequent methamphetamine injection (≥daily vs. <daily), frequent midazolam injection (≥daily vs. <daily), binge drug use (yes vs. no), use of drugs in combination (yes vs. no), injecting with others on a frequent basis (≥25 % of the time vs. <25 % of the time), enrollment in voluntary drug treatment (including methadone treatment) (yes vs. no), reporting difficulty accessing sterile syringes (yes vs. no), and receiving antiretroviral therapy (ART) at the time of interview (receiving vs. not receiving). All behavioral variables refer to the previous 6 months unless otherwise indicated. All *p* values were two-sided.

## Results

A total of 650 unique individuals were seen during the study period, of which 127 (19.5 %) HIV-positive individuals with complete data were eligible for this study. Among the 127 individuals included in this study, 25 (19.7 %) were female. The median age was 38 years (interquartile range, 34–43 years). In total, 21 (16.5 %) participants reported that they had lent a used syringe to another person within the 6 months prior to their interview. As indicated in Table 1, factors significantly and positively associated with syringe lending in bivariable analyses included the following: daily methamphetamine injection (odds ratio (OR) = 10.20, 95 % CI, 2.08–53.60), daily midazolam injection (OR = 3.14, 95 % CI, 1.13–8.72), use of drugs in combination (OR = 4.49, 95 % CI, 0.98–41.60), injecting with others on a frequent basis (OR = 4.25, 95 % CI, 1.26–18.30), and not receiving ART (OR = 2.93, 95 % CI, 1.09–7.86).

**Table 1:** Bivariable analysis of factors associated with syringe lending among a community-recruited sample of HIV-positive PWID in Bangkok, Thailand (*n* = 127)

Characteristic	Syringe lending^a^		Odds ratio (95 % CI)	*p* value
	Yes 21 (16.5 %)	No 106 (83.5 %)		
Age				
<38 years	11 (52.4)	52 (49.1)	1.14 (0.45–2.92)	0.781
≥38 years	10 (47.6)	54 (50.9)		
Gender				
Male	19 (90.5)	83 (78.3)	0.38 (0.04–1.78)	0.246
Female	2 (9.5)	23 (21.7)		
Education level				
<Secondary education	11 (52.4)	42 (39.6)	1.68 (0.65–4.29)	0.279
≥Secondary education	10 (47.6)	64 (60.4)		
Incarceration^a^				
Yes	3 (14.3)	13 (12.3)	1.19 (0.20–4.99)	0.728
No	18 (85.7)	93 (87.7)		
Heroin injection frequency^a^				
≥Daily	2 (9.5)	15 (14.2)	0.64 (0.07–3.14)	0.736
<Daily	19 (90.5)	91 (85.8)		
Methamphetamine injection frequency^a^				
≥Daily	6 (28.6)	4 (3.8)	10.20 (2.08–53.59)	0.001
<Daily	15 (71.4)	102 (96.2)		
Midazolam injection frequency^a^				
≥Daily	15 (71.4)	47 (44.3)	3.14 (1.13–8.72)	0.023
<Daily	6 (28.6)	59 (55.7)		
Binge drug use^a^				
Yes	11 (52.4)	34 (32.1)	2.33 (0.90–6.01)	0.076
No	10 (47.6)	72 (67.9)		
Use of drugs in combination^a^				
Yes	19 (90.5)	72 (67.9)	4.49 (0.98–41.55)	0.037
No	2 (9.5)	34 (32.1)		
Injected with others on a frequent basis^a^				
≥25 % of the time	17 (81.0)	53 (50.0)	4.25 (1.26–18.33)	0.015
<25 % of the time	4 (19.0)	53 (50.0)		
Enrolled in voluntary drug treatment (including methadone)^a^				
Yes	8 (38.1)	38 (35.8)	1.10 (0.42–2.89)	0.845
No	13 (61.9)	68 (64.2)		
Difficulty accessing sterile syringes^a^				
Yes	0 (0.0)	5 (4.7)		0.590
No	21 (100.0)	101 (95.3)		
Receiving antiretroviral therapy^b^				
Receiving	7 (33.3)	63 (59.4)		
Not receiving	14 (66.7)	43 (40.6)	2.93 (1.09–7.86)	0.028

*PWID* people who inject drugs

^a^Denotes events/activities during the 6 months prior to the interview

^b^Denotes events/activities at the time of the interview

## Discussion

A high prevalence of used syringe lending was observed among our sample of HIV-positive PWID in Bangkok, with 16.5 % of participants reporting syringe lending in the past 6 months. Individuals who reported syringe lending were more likely to report high intensity drug use (i.e., daily injection of methamphetamine or midazolam), polysubstance use, frequently injecting with others, and not receiving ART.

It is particularly concerning that individuals who lent syringes were more likely to be untreated for HIV, given the known benefits of ART in preventing HIV transmission [6]. This finding may suggest that individuals receiving ART may have greater access to NSPs and/or education on safer injecting practices secondary to their engagement in HIV care, compared to PWID not receiving ART, who may lack access to such education or services. Furthermore, HIV testing rates remain low in the Asia-Pacific region, with approximately 20 % of PWID having been previously tested [7] and approximately 13 % of PWID avoiding HIV testing in one study in Thailand [8]. Similarly, PWID in Thailand demonstrate considerably low rates of ART coverage, with only 2 % of HIV-positive PWID estimated to have ever accessed ART [4], compared to a reported ART coverage rate of 65 % among all eligible people living with HIV in Thailand [9]. Given Thailand’s system of universal healthcare that provides ART for free or at a reduced cost [10], the comparatively lower rates of ART uptake among PWID may be attributable to the Thai government’s aggressive drug law enforcement strategies that may serve as barriers or deterrents for PWID to access health care [11, 12]. In this context, the lower odds of receiving ART among PWID who lent syringes in this study may suggest that aggressive enforcement may be shaping HIV risk behavior and ART uptake by discouraging access to HIV treatment and prevention programs. Therefore, targeted efforts to reduce the public health burden of aggressive drug law enforcement strategies (e.g., compulsory drug detention) within this setting should occur alongside initiatives to scale up evidence-based public health interventions including HIV testing, ART, peer-based HIV education, and NSPs, as described below [13]. Strategies to address these gaps are currently underway in Thailand, including plans to provide ART irrespective of CD4 count and harmonize health insurance schemes for greater service deliver efficiency [14]. Other interventions to enhance access to and implementation of early ART initiation should be also supported for HIV-positive PWID in Thailand. Finally, given the high prevalence (85–90 %) of HIV and hepatitis C (HCV) co-infection among PWID in Thailand [15, 16], as well as the greater transmissibility of HCV compared to HIV, these findings warrant further research on syringe lending behaviors among HCV-infected PWID and increased HCV testing, treatment, and education for PWID in Thailand.

The associations between syringe lending and high intensity drug use are consistent with previous literature on PWID in Thailand. Specifically, the association between daily methamphetamine injection and syringe lending in this study is consistent with a 2011 study that found that frequent methamphetamine injection was positively and independently associated with syringe sharing among Thai PWID [17]. Additionally, previous studies have found associations between syringe sharing and polysubstance use [18]. Given that midazolam is commonly used in combination with other drugs, the observed association between daily midazolam injection and syringe lending in this study may reflect the high prevalence of polysubstance use among midazolam injectors in Bangkok [19, 20]. Finally, our finding that syringe lending was associated with frequent injection with others complements previous associations found between frequent injection with others and syringe borrowing among Thai PWID [21].

These findings add to the growing body of literature pointing to the high prevalence of risk behaviors for HIV and other infectious diseases (e.g., hepatitis C) among Thai PWID and the importance of harm reduction programs as an essential response to these epidemics [22]. In particular, NSPs have been shown to dramatically reduce the prevalence of high-risk injection behaviors, as evidenced by a decrease in syringe sharing among PWID by more than 38 % due to the distribution of sterile injection equipment in one Canadian setting [23]. Furthermore, NSPs have been shown to prevent HIV and hepatitis C transmission among PWID [24]. Previous studies have highlighted the various difficulties that Thai PWID face in accessing sterile syringes, including distance from NSPs, limited pharmacy hours, and being refused syringes at pharmacies [21]. In order to reduce the harms associated with injection drug use, particularly the risk of HIV and other infectious disease transmission, urgent action is needed to address these barriers and expand access to NSPs in Thailand.

This study has several limitations. First, due to the cross-sectional study design, we were unable to determine temporal relationships between the explanatory variables and the outcome. Thus, we are unable to infer causation from this observational study. Additionally, because we observed only 21 participants reporting syringe lending, we were unable to conduct multivariable analyses to examine the independent relationships between the explanatory variables and the outcome. Therefore, the associations observed in the present study should be further examined through multivariable, longitudinal analyses with a larger sample size. Third, the data collected were self-reported, including HIV serostatus, and may be subjected to reporting biases such as socially desirable reporting and recall bias. However, self-reported data are commonly used in observational studies involving PWID and have been repeatedly found to be valid [25, 26]. Nevertheless, it is possible that the use of self-reported serostatus in this study may confound the association between syringe lending and enrolment in antiretroviral therapy, such that individuals who are on ART may more accurately report their HIV serostatus compared to individuals not receiving ART, who may not have been formally tested for HIV. Fourth, given that the study sample was not randomly selected, the study findings may not be generalizable to Thai PWID or PWID in other settings. Finally, we did not collect data on number of injection partners; however, given past research highlighting the potential influence of social networks on syringe sharing behaviors among PWID [24, 27], this is an area that would benefit from further investigation.

In sum, 16.5 % of our sample of HIV-positive PWID in Bangkok reported used syringe lending in the 6 months prior to being interviewed. This high-risk behavior was associated with high intensity drug use, polysubstance use, injecting with others on a frequent basis, and not receiving ART. These findings emphasize the need to expand evidence-based public health interventions for HIV-positive PWID in Thailand, particularly through the expansion of early ART, NSPs, and peer-based HIV education.

## Abbreviations

AIDS: acquired immune deficiency syndrome
ART: antiretroviral therapy
CI: confidence interval
HIV: human immunodeficiency virus
PWID: people who inject drugs
NSP: needle and syringe program
OR: odds ratio
